# Different amounts of moderate to vigorous physical activity and change in glycemic variability in adolescents with type 1 diabetes: is there dose-response relationship?

**DOI:** 10.20945/2359-3997000000254

**Published:** 2020-06-05

**Authors:** Denise Barth Rebesco, Suzana Nesi França, Valderi Abreu de Lima, Neiva Leite, Leandro Smouter, William Cordeiro de Souza, William Ricardo Komatsu, Luis Paulo Gomes Mascarenhas

**Affiliations:** 1 Programa de Pós-Graduação Interdisciplinar em Desenvolvimento Comunitário Departamento de Educação Física Universidade Estadual do Centro-Oeste Irati PR Brasil Programa de Pós-Graduação Interdisciplinar em Desenvolvimento Comunitário. Departamento de Educação Física, Universidade Estadual do Centro-Oeste (Unicentro), Irati, PR, Brasil; 2 Unidade de Endocrinologia Pediátrica Departamento de Pediatria Universidade Federal do Paraná Curitiba PR Brasil Unidade de Endocrinologia Pediátrica, Departamento de Pediatria, Universidade Federal do Paraná (UFPR), Curitiba, PR, Brasil; 3 Departamento de Educação Física Universidade Federal do Paraná Curitiba PR Brasil Departamento de Educação Física, Universidade Federal do Paraná (UFPR), Curitiba, PR, Brasil; 4 Prefeitura Municipal de Três Barras Três Barras SC Brasil Prefeitura Municipal de Três Barras, Três Barras, SC, Brasil; 5 Divisão de Endocrinologia Departamento de Medicina Universidade Federal de São Paulo São Paulo SP Brasil Divisão de Endocrinologia, Departamento de Medicina, Universidade Federal de São Paulo (Unifesp), São Paulo, SP, Brasil

**Keywords:** Moderate to vigorous physical activity, glycemic variability, type 1 diabetes mellitus

## Abstract

**Objective:**

To identify the level of physical activity and glycemic variability of adolescents with type 1 diabetes mellitus and to compare glycemic variability on days with different amounts of moderate to vigorous physical activity (MVPA).

**Subjects and methods:**

A sample of 34 subjects aged 10 to 15 years, 18 (52.94%) female; age: 13.04 ± 1.94; HbA1c: 9.76 ± 1.51. Physical activity was measured by wGT3X accelerometer. The glucose data were obtained using continuous glucose monitoring, and the following glycemic variability measures were calculated: standard deviation (SD), low blood glucose index (LBGI), high blood glucose index (HBGI), mean amplitude of glycemic excursions (MAGE), glycemic risk assessment in diabetes equation (GRADE) and coefficient of variation (CV). The most and least active days (the days with greater and lesser time dedicated to physical activities of moderate to vigorous intensity, respectively) were identified. In addition, based on the whole period of accelerometer use, daily means of time spent in MVPA were identified among participants, who were then divided into three groups: up to 100 minutes; from 101 to 200 minutes and above 201 minutes. Then, the measures of glycemic variability were compared among the most and least active days and among the groups too.

**Results:**

The amount of MVPA was significantly different between the days evaluated (237.49 ± 93.29 vs. 125.21 ± 58.10 minutes), but glycemic variability measures did not present a significant difference.

**Conclusion:**

Despite the significant differences in the amount of MVPA between the two days evaluated, the glycemic variability did not change significantly. Arch Endocrinol Metab. 2020;64(3):312-8

## INTRODUCTION

The American Diabetes Association ( 1 ) recommends strategies to improve the lifestyle of adolescents with type 1 diabetes mellitus (T1DM), such as balanced diet and regular physical activity to optimize glycemic control. The International Society for Pediatric and Adolescent Diabetes likewise encourages young people with T1DM to engage in physical activity programs ( 2 ). ADA recommendations for children and adolescents with T1DM include 60 minutes daily (or more) of aerobic activities of moderate to vigorous intensity ( 1 ).

Although it is strongly recommended, physical activity is not always part of the routine of adolescents with T1DM. Some studies suggest that a large proportion of these adolescents do not reach 60 minutes daily of moderate to vigorous physical activity (MVPA) ( 3 ) and tend to be less active than their healthy counterparts ( 4 ). In addition, adolescents with T1DM appear to have reduced aerobic capacity when compared to healthy controls and similar in anthropometric state ( 5 ). Aerobic capacity is an important aspect to be taken into consideration in the treatment of adolescents with T1DM, since better aerobic capacity indexes can predict better glycemic control ( 6 ).

Glycemic control, analyzed by HbA1c, and its associations with physical activity has been studied and presents contradictory results in the literature, such as: better glycemic control associated with MVPA ( 7 ) or non-association between the two variables in a group of patients with T1DM who underwent a 12-weeks training program, where they performed aerobic or resistance exercises ( 8 ). Lack of correlation is probably explained by multiple confounding factors such as age, sex, pubertal stage, body composition, diabetes duration, insulin dose, method of insulin administration, ethnicity, and socioeconomic status, which should be taken into account in cross-sectional or intervention studies ( 7 ).

Evidence about HbA1c has led it to be considered one of the most important tools for monitoring glycemic control. However, a recent study shows that patients with long-standing T1DM may show higher blood glucose fluctuations compared to newly diagnosed patients, regardless of the HbA1c ( 9 ). Other work suggests that hyperglycemia and oscillating glucose levels resulting from extreme glucose variation may be associated with endothelial dysfunction and many of the long-term deleterious complications in T1DM ( 10 ). However, HbA1c cannot provide information on daily glucose variability, which is a critical problem in efforts to improve the health of patients with T1DM ( 11 ).

Thus, in order to explore the associations between physical activity and glycemic control, we aimed to identify the level of physical activity and glycemic variability of adolescents with T1DM, as well as to compare glycemic variability on days with different MVPA amounts.

## SUBJECTS AND METHODS

### Design and participants

The present cross-sectional descriptive study evaluated 34 adolescents with T1DM, who attended the diabetes outpatient clinic of the Pediatric Endocrinology Unit of Federal University of Parana School Hospital, Curitiba, PR, Brazil. Participants were included if they had been diagnosed with diabetes for at least 12 months, were aged between 10 and 15 years and did not have any diabetes-related comorbidities. Parental/guardian consent and adolescent assent were obtained for all participants. The present study was approved by the Research Ethics Committee of the Midwestern Parana State University (Unicentro), Irati, PR, Brazil, under decision number 1.202.475.

The patients were instructed to note their daily insulin dose and record their food intake during the intervention period. In addition to the food logs that were used to calculate daily carbohydrate intake, participants provided photos of home measurements to maximize record accuracy. The quantitative analysis of food intake was performed using the ADS Nutri^®^ diet analysis software, which has a database with more than 3,000 registered foods. The Brazilian Food Composition Table was selected as data sources ( 12 ). The insulin therapy of evaluated was not altered. They were treated with multiple daily injection with long-acting (Glargine) and ultrarapid-acting insulin (Aspart and Lispro).

Tanner’s criteria were used to assess biological maturation ( 13 ). To evaluate maturation, the participant’s self-assessment procedure was used. Images pre-established by Tanner were presented to the subjects, and participants indicated the stage (I, II, III, IV or V) which best described them. The participants were then regrouped according to Tanner stages into the following categories: pre-pubertal (stage I); puberty (stages II, III and IV); and post-pubertal (stage V).

Data on the insulin units applied and the daily intake of carbohydrates in grams, together with the biological maturation data, were considered in calculations and analyses.

### Anthropometric evaluation and blood analysis

Anthropometric measurements were collected using the techniques described by Lohman (1992) ( 14 ). Height, evaluated in centimeters at the end of maximal inspiration, was measured with a portable vertical stadiometer (WCS^®^, Brazil) to the nearest 0.1 cm. Body mass was measured using a portable digital scale (Filizola^®^, Brazil), in kilograms (kg). The body mass index (kg/m^2^) was then calculated by dividing the body mass by the height squared, and converted to BMI z score. Blood was collected by venipuncture and analyzed by TurbiClin immunoturbidimetric test (São Paulo - Brazil), for the assessment of HbA1c.

### Moderate to vigorous physical activity and sedentary behavior

The accelerometer device (Actgraph wGT3X) was worn on the waist and stored data regarding the daily routine of patients’ physical activity for five days (they did not any structured and controlled physical activities, only maintained their daily routines). Those with at least three days of valid activity data, regardless of the day of the week, were included in the analyses. A valid day was defined as ≥ 600 minutes of use time ( 15 ).

Subsequently, the data were exported through specific software (ActiLife version 6.11.2) and the most and least active days were identified. Most and least active days were defined as those with greatest and least time dedicated to physical activities of moderate to vigorous intensity, respectively. In addition, based on the whole period of accelerometer use, daily means of time spent in moderate to vigorous physical activity (MVPA) were identified among participants. As most of them remained from zero to 300 minutes in this intensity, they were then divided into three groups: up to 100 minutes; from 101 to 200 minutes and above 201 minutes. The day that the device was delivered to the adolescent and the day it was returned to the evaluators were excluded from this analysis. To identify the time spent in MVPA and time in sedentary behavior, the cut-off points suggested by Freedson and cols. (2005) ( 16 ) were applied.

### Glycemic variability

Interstitial glucose values were obtained using a real-time continuous glucose monitor (CGM) (Guardian^®^RT, Medtronic, Minimed). A CGM sensor was inserted subcutaneously in the patient’s lumbar region, and collected interstitial glucose measurements every five minutes for five days. Participants were blinded to their glucose values and could not change their regular behavior patterns based on real-time glucose monitoring. Then the data were used to calculate the following glycemic variability measures: low blood glucose index (LBGI), high blood glucose index (HBGI) ( 17 ); mean amplitude of glycemic excursions (MAGE) ( 18 ); and glycemic risk assessment in diabetes equation (GRADE) ( 19 ). The standard deviation (SD) was also used. This measure of variability, is widely used in the evaluation of glycemic profiles, and demonstrates how much variation or dispersion exists around the mean ( 20 ). All glycemic variability measurements were calculated using EasyGV software ( 20 ) and then the data compared between most and least active days.

Moreover, the coefficient of variation (CV) was calculated and used in the comparisons cited above. The CV (which is the SD divided by the mean) is the main parameter of glycemic variability according to International Consensus on Use of Continuous Glucose Monitoring Diabetes ( 21 ).

### Data analysis

Data were collected using Microsoft^®^ Office Excel software, version 2010 (Redmond, WA, USA) and analyzed using IBM^®^ SPSS software – Statistical Package for Social Sciences, version 21 (Chicago, IL, USA). Mean and standard deviation were used for descriptive statistics. In order to verify if there was difference between the most and least active days, as well as differences in the GV between the two days, Student’s t test was used. ANOVA test was used to verify if there was difference in the glycemic variability between the groups with different daily averages of MVPA. All tests adopted α of 0.05

## RESULTS

Thirty-four individuals (16 males and 18 females) participated in this study. Participants presented similar age, weight, height, and BMI-Z score. The general characteristics of the individuals are described in table 1 .

**Table 1:** Sample characteristics

	Total n = 34	Male n = 16	Female n = 18	p
Age (years)	13.04 ± 1.94	13.21 ± 2.01	12.90 ± 1.91	0.65
Time T1DM diagnosis (years)	5.93 ± 3.78	5.49 ± 3.57	6.38 ± 4.04	0.51
Body mass (kg)	47.85 ± 13.16	46.03 ± 13.66	49.38 ± 12.89	0.46
Height (cm)	153.90 ± 13.19	153.43 ± 15.59	154.31 ± 11.21	0.84
BMI z score	0.34 ± 0.87	0.15 ± 0.84	0.49 ± 0.88	0.25
HbA1c (%)	9.76 ± 1.51	9.89 ± 1.69	9.64 ± 1.37	0.64
Time in hypoglycemic range (%)	1.60 ± 2.07	1.79 ± 1.98	1.43 ± 2.18	0.61
Time in range (%)	39.21 ± 14.33	42.34 ± 16.36	36.44 ± 12.05	0.23
Time in hyperglycemic range (%)	59.17 ± 15.15	55.85 ± 16.66	62.11 ± 13.46	0.23
Carbohydrate/day (g)	241.31 ± 56.71	257.04 ± 61.46	228.35 ± 50.66	0.16
Insulin (UI/day)	48.26 ± 16.77	48.16 ± 19.91	48.36 ± 14.01	0.97
Pre-pubertal	6 (17.64%)	3 (18.75%)	3 (16.66%)	0.50
Puberty	16 (47.06%)	8 (50.00%)	8 (44.45%)	-
Post-pubertal	12 (35.30%)	5 (31.25%)	7(38.89%)	-

Notes: Carbohydrate/day (g), insulin (UI/day) and pubertal stage, besides characterizing the sample, were considered in calculations and analyses.

Table 2 shows the comparison between the most and least active days of the sample, a significant difference is observed.

**Table 2:** Comparison between the most and least active days

	Most active day	Least active day	P
MVPA (min)	237.49 ± 93.29*	125.21 ± 58.10	0.001

*p < 0.001.

Table 3 presents the comparison between the GV measures for the most and least active days. We can observe there was no difference between the two days.

**Table 3:** Comparison between GV measures (mmol/L) between the most and least active days

	Most active day	Least active day	Variation	p
MEAN	11.56 ± 2.43	11.50 ± 2.21	0.06 ± 2.60	0.90
SD	3.62 ± 1.06	3.65 ± 1.20	-0.03 ± 1.39	0.91
LBGI	2.22 ± 4,92	1.61 ± 1.84	0.60 ± 4.82	0.50
HBGI	17.66 ± 7.40	17.43 ± 7.64	0.23 ± 9.54	0.89
GRADE	13.66 ± 5.48	13.62 ± 5.89	0.04 ± 6.13	0.97
MAGE	9.45 ± 3.29	9.62 ± 3.04	-0.17 ± 3.68	0.80
CV	32.84 ± 12.06	32.13 ± 9.15	-0.70 ± 12.25	0.75

Notes: SD: standard deviation; LBGI: Low Blood Glucose Index; HBGI: High Blood Glucose Index; MAGE: Mean amplitude of glycemic excursions; GRADE: Glycemic Risk Assessment in Diabetes Equation; CV: Coefficient of variation.

Table 4 presents the comparison of GV between groups with different average amounts of moderate to vigorous physical activity.

**Table 4:** GV Comparison (mmol/L) between groups

	Up to 100 (min)	101 a 200 (min)	Above 201 (min)	F*	p
MEAN	12.80 ± 0.30	11.69 ± 1.72	12.16 ± 1.86	0.49	0.61
SD	3.68 ± 0.39	4.23 ± 0.87	4.63 ± 1.03	1.06	0.36
LBGI	1.10 ± 0.33	2.56 ± 2.21	3.85 ± 1.03	0.55	0.58
HBGI	20.13 ± 0.07	18.37 ± 5.98	21.30 ± 6.16	0.71	0.49
GRADE	16.78 ± 0.18	13.60 ± 4.68	15.10 ± 4.19	0.68	0.52
MAGE	8.79 ± 2.77	9.56 ± 2.51	9.77 ± 2.54	0.12	0.88
CV	28.79 ± 3.75	36.35 ± 4.67	38.47 ± 8.70	1.94	0.16

Notes: min: minutes; F*: F-statistics; SD: standard deviation; LBGI: Low Blood Glucose Index; HBGI: High Blood Glucose Index; MAGE: Mean amplitude of glycemic excursions; GRADE: Glycemic Risk Assessment in Diabetes Equation. CV: Coefficient of variation.

## DISCUSSION

Results were similar between males and females ( Table 1 ), reflecting inadequate glycemic control with mean HbA1c above the recommended value ( 1 ). Some studies have shown different results when the patients are compared by sex, with worse glycemic and metabolic control among the females, who according to the same studies are more prone to complications in adulthood ( 22 ) ( 23 ). Perhaps what contributed to the similarities between males and females in relation to glycemic control in this sample was the homogeneity regarding age, weight, height, BMI-Z score and maturational stage.

Maintaining adequate levels of HbA1c is important because values greater than 7.5% represent an increased risk for long-term health complications ( 24 ). Some habits, such as adequate diet and regular practice of physical activity, may interfere positively in the treatment of individuals with T1DM, improving glycemic control ( 25 ). In this study, glycemic control was also analyzed by glucose variability, using the following measures: SD, LBGI, HBGI, GRADE, MAGE and CV. Except for coefficient of variation (CV), there is no reference range for GV in subjects with diabetes. For CV, stable glucose levels are defined as a CV < 36% ( 21 ).

In the table 1 time in range is presented, the values suggest that the sample does not achieve the target range proposed by the International Consensus on Time in Range (>70%) ( 26 ). The values still suggest a high percentage of time in hyperglycemic range, this information can be corroborated by high values of HBGI presented in tables 3 and 4 .

Besides identifying glycemic variability, through the measures mentioned above, the present study compared it between the most active day and the least active day of the participants. In order to obtain glucose data a CGM was used, which according to Lachin and cols. (2017) ( 27 ) is considered the gold standard to capture variability. Many studies have used CGM to investigate glycemic variability, as well as to compare or verify its association with several parameters. To the best of our knowledge there is no study that has evaluated glycemic variability during the daily routine of adolescents with T1DM and performed a comparison, taking into account most or least active days.

However, among the investigations about glycemic variability it is possible to observe studies with different approaches. Some evaluated glycemic variability in relation to insulin treatment, in order to understand if the changes in the hormone administration could influence it. Lucchesi and cols. (2012), when evaluating the effects of a treatment that mixed two types of insulin (Lispro and Glargine) did not observe any difference in glycemic variability ( 28 ). Similar results were found by Iga and cols. (2017), where glycemic variability was similar among individuals treated with different types of insulin (degludec and glargine) ( 29 ). In the present study, a significant increase in the amount of MVPA represented small changes in the glycemic variability.

The increase in the MVPA amount observed in this study was approximately 90% between the two days analyzed ( Table 2 ), however the results presented in table 3 suggest that, although there were significant differences in the MVPA amount, glycemic variability was not significantly altered. This finding strengthens the claim that physical activity can be safely included into the diabetic patient routine ( 2 ).

In the present study, patients were divided in three groups according to the mean daily time spent on activities of moderate to vigorous intensity, however the glycemic variability did not present difference between groups ( Table 4 ). This finding suggests that there is no dose response relationship between MVPA and glycemic variability, since the increase in the amount of physical activity did not lead to an increase in glycemic variability. On the other hand, the results found in study by Manohar and cols. (2012) suggest lower glycemic variability after low intensity physical activity, in this study the postprandial glycemic excursions were analyzed in individuals who did or did not engage in physical activity, as well as in patients with T1DM and healthy controls ( 30 ).

Besides the glycemic variability measures, which did not significantly alter between days ( Table 3 ) and groups ( Table 4 ) there was an important difference in LBGI. Although not statistically significant, the numerical difference is great. The sample size can affect this significance.

Regarding the time spent in MVPA ( Table 2 ), even on the day considered least active, participants in this study reached the recommendation to accumulate at least 60 minutes of MVPA. On the other hand, in the study by Maggio and cols. (2010), only 38% of evaluated children with T1DM reached this goal, this number increased to 60% in the control group ( 4 ). The evaluation of the time spent in MVPA is especially important for adolescents with T1DM, it is suggested that those who spend more time in MVPA demonstrate better glycemic control ( 3 ).

When promoting physical activity as healthy behavior, it is important to guide adolescents with T1DM regarding their effects on glycemic variability. Through this study it is suggested that acutely assessed glycemic variability exists and it is influenced by the amount of physical activity up to a certain point. On the other hand, future studies are necessary to determine the influence of chronic physical activity on glycemic variability measures, since there are no studies that verify the influence of a program of regular physical activity on glycemic variability of T1DM adolescents.

Finally, concerning strengths and limitations, this report has a clear clinical value as it shows that moderate to vigorous physical activity does not worsen glycemic variability in adolescents with type 1 diabetes. This information highlights that patients with type 1 diabetes should not avoid physical activity because of possibly “messing up” their control. However, we don’t know if would this result be the same in a group of patients with better baseline glycemic control. In addition, the sample size cannot permit us to reach a firm conclusion.

In conclusion, although there were significant differences in the percentage of moderate to vigorous physical activity between the two days, the glycemic variability did not change significantly.
